# Hot Compression Behavior and Processing Maps of 6063 Aluminum Alloy Under Medium Strain Rate

**DOI:** 10.3390/ma18112510

**Published:** 2025-05-27

**Authors:** Zhenhu Wang, Qincan Shen, Shuang Chen, Lijun Dong, Erli Xia, Pengcheng Guo, Yajun Luo

**Affiliations:** 1 School of Mechanical Engineering, Hunan Institute of Engineering, Xiangtan 411104, China; 21109@hnie.edu.cn (Z.W.); luoyajun@hnie.edu.cn (Y.L.); 2 College of Mechanical and Vehicle Engineering, Hunan University, Changsha 410082, China; 3 Jiangnan Industries Group Co., Ltd., Xiangtan 411200, China; 4 School of Intelligent Manufacturing and Mechanical Engineering, Hunan Institute of Technology, Hengyang 421002, China

**Keywords:** 6063 aluminum alloy, hot compression, hot processing map, microstructure observation

## Abstract

A hot compression test was conducted across a range of temperatures (350, 400, 450, and 500 °C) and varying strain rates (0.001–10 s^−1^) to explore the hot compression behavior of the 6063 alloy. Hot processing maps were obtained based on the stress–strain curves. Optimal processing parameters were identified as residing within the intervals of (470–500 °C, 0.01–0.1108 s^−1^), achieving a maximum dissipation efficiency of 0.4, which is of great importance for perfecting hot processing. The microstructure evolution was characterized using an optical microscope and a transmission electron microscope. The initial grains were elongated under compressive deformation, and the density of dislocation rose with increasing strain rate and decreasing temperature. Dynamic recovery serves as the main dynamic softening mechanism during hot compression.

## 1. Introduction

Aluminum alloys have excellent specific strength, relatively low density, outstanding formability, and exceptional corrosion resistance [1,2]; they are a prime candidate for replacing ferrous materials in the production process in the aircraft, space, military, automotive, and electronic industries [3,4]. Utilizing aluminum alloys can result in significant weight reduction, consequently decreasing CO_2_ emissions and contributing to the goals of environment protection [5]. The 6000-series alloy exhibits superior comprehensive properties—for instance, excellent mechanical properties, favorable corrosion resistance, and prime forming ability—and is extensively utilized in many fields, including airplane, vehicle, and railway transportation [6]. The mechanical properties and servicing performance of 6000-series alloys vary with the different compositions of Mg and Si elements. Meanwhile, the Mg/Si ratio exerts a massive impact on the evolution of precipitates [7], profoundly influencing the properties of the material. The precipitate will impede the dislocation movement and will affect the recrystallization significantly. The 6063 aluminum alloy is a typical 6000-series alloy; the contents of Si and Mg are 0.2–0.6% and 0.45–0.9%, respectively [8]. Therefore, its strength is slightly less than that of 6061 and 6082 alloys, but it can obtain a comparable strength after plastic deformation. At the same time, the 6063 alloy has a better forming ability and a distinctly superior finishing quality [9]. As a result, it is frequently chosen for fabricating profiles with complex sectional shapes, particularly for structural and load-bearing components in the aerospace and automotive industries. The 6063 alloy components are commonly processed through various thermal forming methods: extrusion, rolling, and forging at an elevated temperature [10,11,12]. An appropriate processing technique is essential for ensuring both the workability and service performance of the material; therefore, processability is a crucial factor for predicting the hot formability of metallic materials.

Researchers have introduced the concept of processing maps based on the dynamic material model [13,14]. Yu et al. [15] explored the hot compression behavior of the Al-Mg-Si-Ce-B alloy and developed processing maps, which identified the optimal deformation parameters as 510–550 °C/0.03–0.04 s^−1^. Ghosh et al. [16] presented an in-depth analysis of the hot compression and workability of an Al-Mg-Si alloy through hot compression conducted within a temperature range of 400–550 °C and a strain rate range of 0.001–1 s^−1^. The processing maps indicated a safe processing domain for proper hot working parameters between 480 and 550 °C and 0.001–0.01 s^−1^. Feng et al. [17] demonstrated that the appropriate forming conditions for the optimal microstructure and properties in Al-Mg-Si alloy are 480–520 °C/0.03–0.2 s^−1^. Processing maps are widely employed to ascertain the proper forming conditions, to regulate the microstructure, and to prevent defects [18,19,20,21,22]. In summary, processing maps are of great importance in the manufacturing process of aluminum alloy components, such as car frames, car bumpers, and aircraft landing gear components.

In the domain of hot working, the microstructural evolution within materials is acutely responsive to the processing strain rate, temperature, and deformation degree. Concurrently, the serving performance of the components profoundly impacts their microstructure [23,24]. Snopiński [25] examined the deformation response of annealed Al-Mg-Si material, revealing that the annealed alloy exhibits lower flow stress. The study identified that both dynamic recovery (DRV) and dynamic recrystallization (DRX) are primary deformation mechanisms. The findings provide significant insights into the hot processing of aluminum alloy. Jin [26] investigated the hot compression response and microstructure mechanism of an Al-Mg-Si alloy. Their research revealed that precipitation has a significant impact on deformation behavior. The findings provide insights into optimizing the processing parameters of the alloy. Feng et al. [17] explored DRX characteristics in an Al-Mg-Si alloy and their subsequent influences on flow stress under various hot deformation conditions. The research identified that higher deformation temperatures and lower strain rates facilitate uniform grain size through DDRX and CDRX mechanisms, which is crucial for achieving superior mechanical properties after solid solution treatment. Zhao et al. [27] comprehensively investigated the hot compression behavior and microstructural development of an Al-Mg-Si alloy. The research identified that DRX, including continuous, discontinuous, and geometric dynamic recrystallization (CDRX, DDRX, and GDRX), was significantly influenced by the Zener–Hollomon parameter (Z), with the proportion of high-angle grain boundaries (HAGBs) and recrystallized grains increasing with temperature and a declining strain rate. Hu et al. [28] investigated the hot compression response of an Mg-Si alloy conducted within 300–500 °C/0.01–5 s^−1^. The research identified that DRV and partial DRX contribute to the high efficiency of power dissipation in specific regions of the processing map. These findings are instrumental for optimizing the manufacturing processes of Al-Mg-Si alloys for automotive applications.

In previous studies, the true strain utilized in the compression tests was typically not large. Nonetheless, under conditions of large strain compression, the microstructure evolution and flow behavior of the alloys are entirely different compared to those observed at lower strains. Deformation parameters profoundly influence achieving the desired final properties of the components. A comprehensive understanding is essential for developing advanced manufacturing techniques and the effective production of high-quality products. In industrial production, 6063 aluminum alloy components are commonly adopted in vehicle components, rail transit, and airplane auxiliary components, which can significantly reduce vehicle weight and improve fuel efficiency. These applications are usually processed through hot plastic deformation at a wide range of temperatures (600–800 K) and large deformations to obtain excellent properties and complex shapes. According to previous studies, when a Mg/Si ratio of 1.48 was chosen, it led to denser precipitation and resulted in better strength [7]. Meanwhile, it was reported that the flow stress of 6063 alloy could be effectively enhanced with Mg content up to 0.651% [8]. Based on the above consideration, in the present work, the Mg content in this work was chosen as 0.65%, along with a Mg/Si ratio of 1.41. Therefore, an in-depth exploration of compression response and microstructure development of 6063 alloy via hot compression testing with large strain is conducted. The aim is to elucidate the deformation mechanisms and to provide a reference for choosing alloy processing parameters and enhancing properties.

## 2. Materials and Experimental Methods

In the present research, a conventional 6063 alloy was designated as the experimental subject. The chemical constituents are detailed in Table 1. The research specimen was characterized as a hot-rolled 10 mm plate.

**Figure 1:**
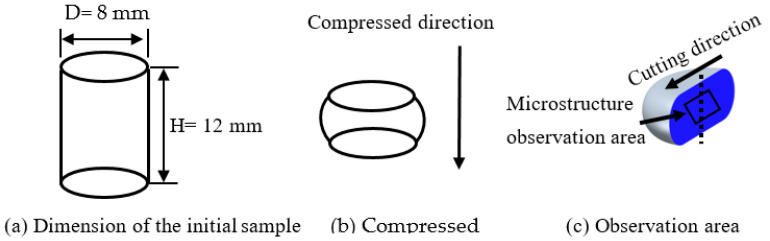
Sample dimension and observation area.

In the present study, cylindrical specimens were machined for subsequent hot compression tests, and their dimensions are displayed in Figure 1a. The diameter and height of the specimen were 8 mm and 12 mm, respectively. The height direction of the specimens was aligned with the rolling direction. A Gleeble-3500 was applied (DSI Corporation, St. Paul, MN, USA), and hot compression was conducted in different strain rates (0.01–10 s^−1^) and temperatures (350–500 °C). The heating rate is 10 °C/s, as the aim temperature was obtained, a 3 min holding was conducted, to ensure homogeneity of temperature. A true strain of 0.75 was targeted for all specimens under test conditions, as shown in Figure 1b. To cut down the effects of friction, a layer of graphite lubricant was interposed between the press head and the samples. Upon completion of each compression test, the specimens were quenched in water immediately to freeze the microstructure for subsequent analysis.

To figure out the influence of compression parameters on microstructural development, optical microscopy (OM) and transmission electron microscopy (TEM) were employed. Following the compression tests, the center position of the sectioned surface was selected for microstructure characterization, as illustrated in Figure 1c. For OM analysis, the samples were mechanically polished using a polishing apparatus (Ortlay, Tianjin, China) and subsequently etched in a solution comprising 4% HBF_4_ and 96% H_2_O. The microstructures were then observed by an optical microscope (Axio Vert A1 CarlZeiss, GmbH, Oberkochen, Germany). For TEM observation, the samples were thinned down to 70 μm by grinding, followed by twin-jet electropolishing with a twin-jet setup (Struers, Shanghai, China) in a solution mixture of 30% HNO_3_ and 70% CH_3_OH at a cryogenic condition of −30 to −20 °C. The TEM observation was conducted on a Tecnai G2 20 microscope (FEI Company, Hillsboro, OR, USA).

## 3. The Construction of Processing Map

A processing map was established upon the dynamic materials model (DMM) theory [29]. The DMM postulates that during the hot processing, the whole power dissipated, denoted as *P*, is bifurcated into two distinct components, which can be articulated as follows [30]:

(1) $$P=\sigma \;\dot{\varepsilon }=\int_{0}^{\dot{\varepsilon }}\sigma \;d\;\dot{\varepsilon }+\int_{0}^{\sigma }\dot{\varepsilon }\;d\;\sigma$$

In this context, σ symbolizes the flow stress, while $\dot{\varepsilon }$ represents the strain rate; *G* signifies the power dissipated through the mechanisms of heating and deformation; *J* denotes the power used by microstructure development processes, including but not limited to DRV, superplastic flow, and DRX.

It is a widely accepted notion that the strain rate substantially influences flow stress during deformation. To investigate the status of energy dissipation, the strain rate sensitivity coefficient (*m*) is initially introduced as a characteristic parameter of the hot processing. The coefficient m can be determined by differentiating the power dissipated between deformation heat and microstructural evolution, as follows [31]:

(2) $$m=\frac{dJ}{dG}=\frac{\partial P}{\partial G}\frac{\partial J}{\partial P}=\frac{\dot{\varepsilon }d\sigma }{\sigma d\dot{\varepsilon }}=\frac{\partial (\ln\sigma )}{\partial (\ln\dot{\varepsilon })}$$

Employing the *m* and σ during the hot processing could be articulated according to the following expression [31]:

(3) $$\sigma =K^{\dot{\varepsilon }m}$$

where *K* is a material-specific constant. By combining Equations (1) and (3), the *J*, which represents the power dissipated due to microstructural changes, can be derived as follows [32]:

(4) $$J=\frac{m}{m+1}\sigma \dot{\varepsilon }$$

Under linear dissipation, the *m* is equated to 1. In such a scenario, the *J*, which corresponds to power dissipated due to microstructural evolution, attains its maximum value.

(5) $$J_{\max}=\frac{1}{2}\sigma \dot{\varepsilon }$$

Hot processing cannot be characterized as involving linear dissipation. The power dissipation efficiency, denoted as *η*, is defined by the actual power dissipated due to the microstructure developed, *J,* to the peak possible power dissipation, *J*_max_, as follows [32]:

(6) $$\eta =\frac{J}{J_{\max}}=\frac{2m}{1+m}$$

The dissipation efficiency under different deformation parameters could be ascertained by applying Equations (2) and (6), thereby enabling the construction of a visual power dissipation map. A definitive relationship exists between *η* and deformation mechanisms, which could provide insights into the hot processing. Consequently, *η* plays a key role in the development of the processing map, as it aids in identifying optimal processing parameters and understanding the underlying material behavior during hot working operations.

Furthermore, to circumvent the inception of cracks and the occurrence of flow localization, a calculation methodology for the instability parameter has been introduced by Prasad and Seshacharyulu [33], which facilitates the identification of unstable domains. This parameter can be articulated mathematically as follows:

(7) $$\xi (\dot{\varepsilon })=\frac{\partial \ln[m/(m+1)]}{\partial \ln\dot{\varepsilon }}+m<0$$

Equation (7) illustrates that *ξ* varies under different processing parameters. By computing *ξ* across different deformation parameters, a visual instability map could be constructed. This map delineates regions with negative *ξ* values, indicating flow instability, within which the hot processing is markedly compromised. Operating within these unstable domains can lead to the alloy microstructure exhibiting localized flow, cracking, and other deformation-related defects. Consequently, it is imperative to avoid these zones during the manufacturing process of structural components to ensure optimal microstructural integrity. Extensive results from the literature have validated the efficacy of the processing map in identifying optimal processing conditions.

## 4. Results and Discussion

### 4.1. Initial Microstructure

**Figure 2:**
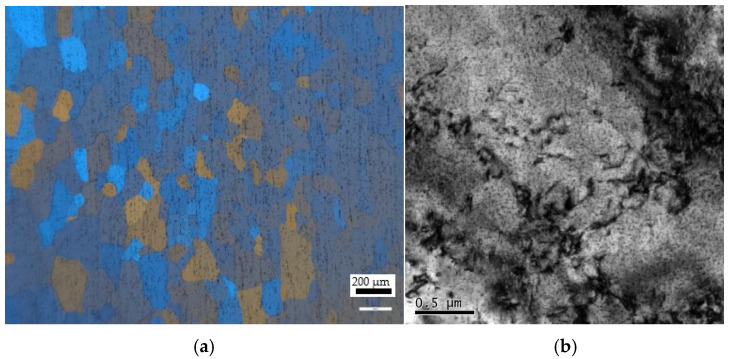
The initial microstructure: (**a**) OM observation; (**b**) TEM observation.

Figure 2 presents the initial microstructure of 6063 aluminum alloy before compression. As shown in Figure 2a, the matrix contains many equiaxed grains, the average size is about 60 μm, and cracks and large second-phase particles were found during hot compression. Figure 2b displays the TEM micrograph, where many dislocations can be observed. Furthermore, it was observed that the Mg_2_Si precipitates with a small size were uniformly distributed in the matrix [7,8].

### 4.2. The True Stress–Strain Curve

**Figure 3:**
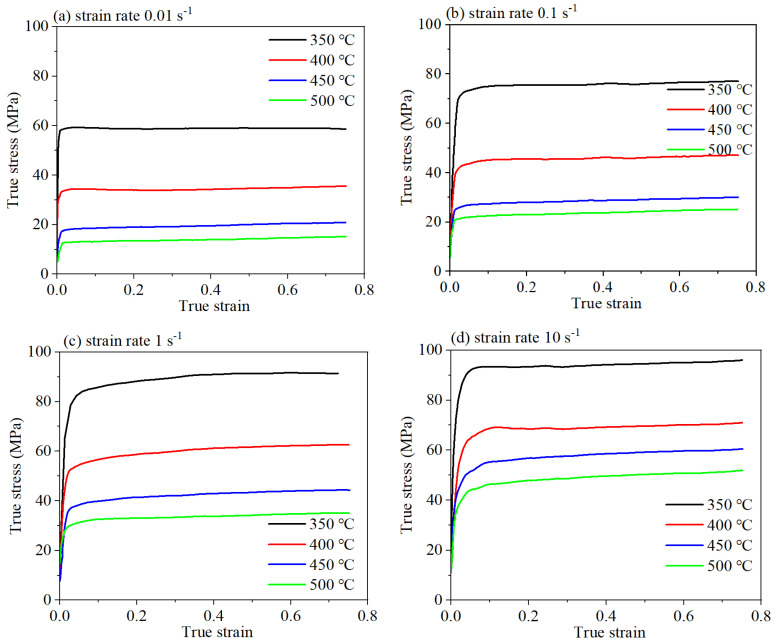
The true stress–strain curves of 6063 alloy: (**a**) 0.01 s^−1^; (**b**) 0.1 s^−1^; (**c**) 1 s^−1^; (**d**) 10 s^−1^.

The true stress–strain curves under varying experimental parameters are shown in Figure 3. Firstly, there is a pronounced increment in flow stress with an increment of strain. Subsequently, the stress surpasses the yield strength rapidly, while the deformation, work hardening, and softening are in a competitive relationship. Dislocation multiplication, driven by deformation, manifests as work hardening, whereas an escalation in internal energy engendering dynamic recovery and recrystallization [34]. At this moment, the rise in dislocation density accounts for the increment in true stress, signifying that work hardening takes precedence over softening effects. Then, the dynamic softening effect intensifies, partially offsetting work hardening effects and consequently slowing down the rate of stress increment. The competition between softening and work hardening results in the stress–strain curve. As the deformation continues, the softening effect is slightly weaker than the hardening effect, culminating in a modest upward trend of stress in the subsequent deformation [35].

Furthermore, Figure 3 elucidates that the stress level of 6063 is dramatically influenced by the compression conditions. The compression temperature exerts an inverse impact on the flow stress. At high temperatures, dislocations within the crystal lattice possess increased kinetic energy, which enables dislocations to surmount energy barriers, leading to their rearrangement and a consequent decrement in stress. Conversely, the strain rate positively impacts stress; at higher strain rates, the motion of dislocation is impeded due to the limited deformation time, resulting in an increment of stress with increasing strain rate. The flow behavior of 6063 alloy is congruent with previous research [36]. Typically, an alloy exhibiting a low flow stress level is easy to deform, and the optimal processing regime is often within the domain of low flow stress. It is plausible to deduce that conditions of higher temperature and lower strain rate are beneficial to obtain optimal deformation parameters.

### 4.3. Processing Map

#### 4.3.1. The Analysis of Strain Rate Sensitivity Factor m

Processing maps are exceedingly beneficial in the realm of hot working operations, providing a comprehensive overview of power dissipation and the localization of instability zones. To harness the full utility of these maps, it is imperative to determine the power dissipation efficiency (*η*) and the associated instability parameter $\xi (\dot{\varepsilon })$. The accurate calculation of these parameters can help accurately evaluate m. The factor *m* is integral to the predictive accuracy of the map. The calculation of *m* is facilitated through curve fitting techniques, with a cubic spline function commonly utilized to correlate the strain rate with the flow stress at a given strain level. This relationship is mathematically formulated as follows. The *m* is derived from the slope of the linear regression between the logarithm of the strain rate and the logarithm of the flow stress [37].

(8) $$\ln\sigma =a+b\ln\dot{\varepsilon }+c{\left(\ln\dot{\varepsilon }\right)}^{2}+d{\left(\ln\dot{\varepsilon }\right)}^{3}$$

**Figure 4:**
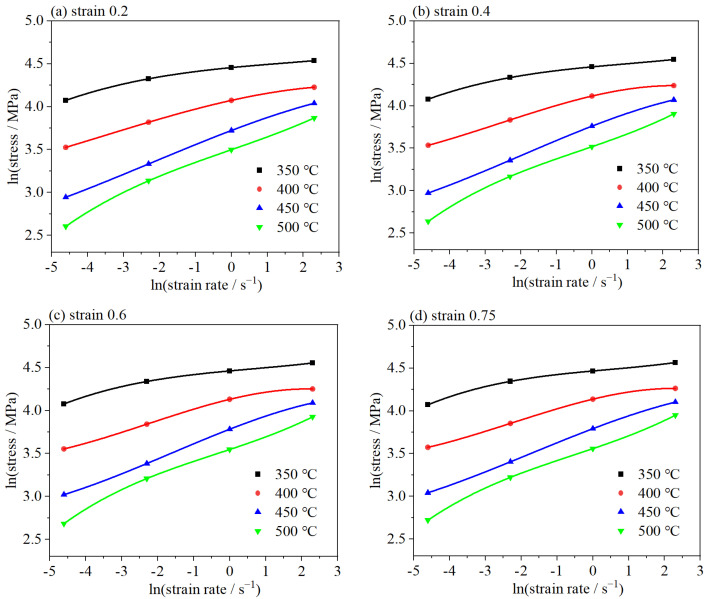
The relationship between ln (stress) and ln (strain rate) at different strains (**a**) strain 0.2; (**b**) strain 0.4; (**c**) strain 0.6; (**d**) strain 0.75.

The parameters a, b, c, and d, which are critical for the mathematical representation of material behavior, are deduced through the curve fitting method. As shown in Figure 4, the interrelation between the lnσ and $\ln\dot{\varepsilon }$ across a range of strains offers insights into the material’s deformation characteristics under different strains.

In accordance with Equations (2) and (8), the subsequent formula could be applied to deduce the strain rate sensitivity factor *m* [37]:

(9) $$m=b+2c\ln\dot{\varepsilon }+3d{\left(\ln\dot{\varepsilon }\right)}^{2}$$

**Figure 5:**
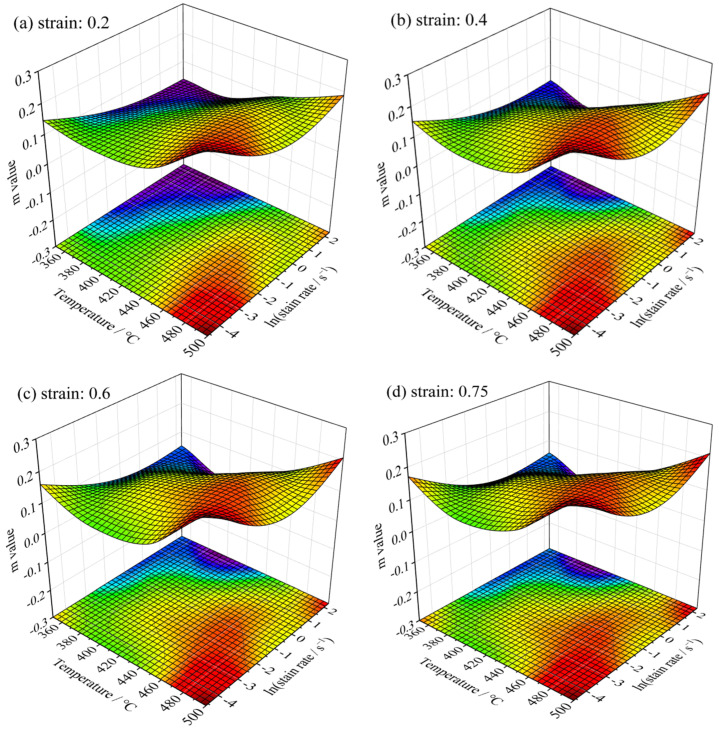
Influence of strain on strain rate sensitivity factor (*m*). (**a**) Strain 0.2; (**b**) strain 0.4; (**c**) strain 0.6; (**d**) strain 0.75. (The blue color represents the lowest *m* value, and the red color highest *m* value).

The *m* achieved across a quartet of distinct temperatures and a range of strain rates, is organized within a 4 × 4 matrix for reference. To manifest the interdependence between the deformation parameters and the m value graphically, interpolation of the m values was conducted, culminating in the generation of a 100 × 100 matrix. Figure 5 presents a three-dimensional representation of the m values, with the *x*-axis corresponding to temperature and the *y*-axis corresponding to strain rate. The visualization reveals that the m values fluctuate non-uniformly in response to variations in deformation parameters. Nevertheless, a comparison of 3D surfaces at varying strains shows a striking similarity.

#### 4.3.2. The Analysis of Power Dissipation Efficiency η

**Figure 6:**
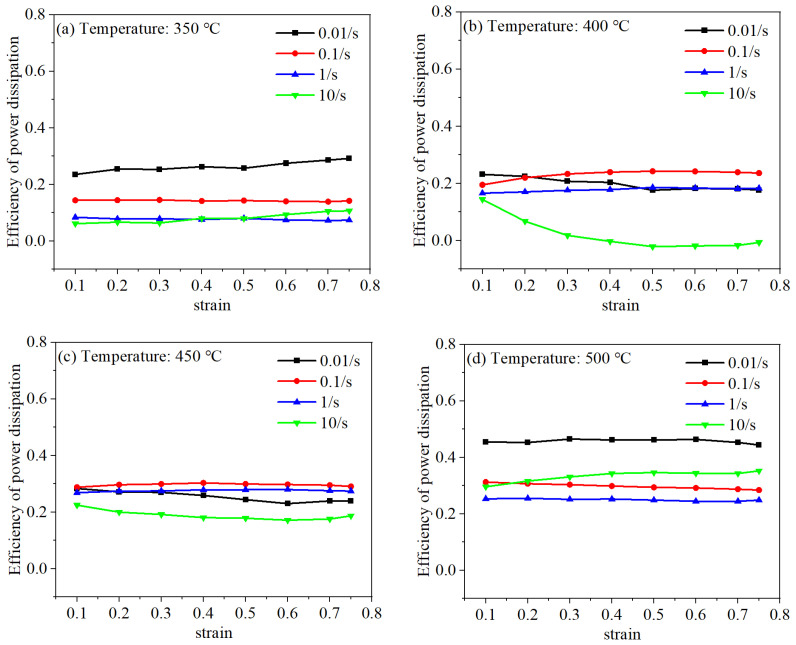
The relationship between power dissipation efficiency (*η*) and strain: (**a**) 350 °C; (**b**) 400 °C; (**c**) 450 °C; (**d**) 500 °C.

Utilizing Equation (6), *η* becomes computable upon the acquisition of strain rate sensitivity factor (*m*) values. However, in order to calculate the parameter *η*, it is assumed that the constitutive relation of the targeted material conforms to a certain model, such as the Johnson–Cook material model, Arrhenius model, and so on. It means that the strain rate sensitivity index *m* is independent of the strain rate, and it should be constant at different strain rates. In fact, the value of m shows a slight change tendency with the variation in deformation conditions. Thus, to some extent, the computed power dissipation efficiency shows a tiny discrepancy with the actual value. Figure 6 displays the relationship between *η* and the applied strain. It is observable that *η* exhibits a pronounced dependency on strain at strain rates of 0.01 and 10 s^−1^. Conversely, at strain rates of 0.1 and 1 s^−1^, the sensitivity of *η* to strain is markedly suppressed. Additionally, an inverse relationship is noted between *η* and the strain rate.

**Figure 7:**
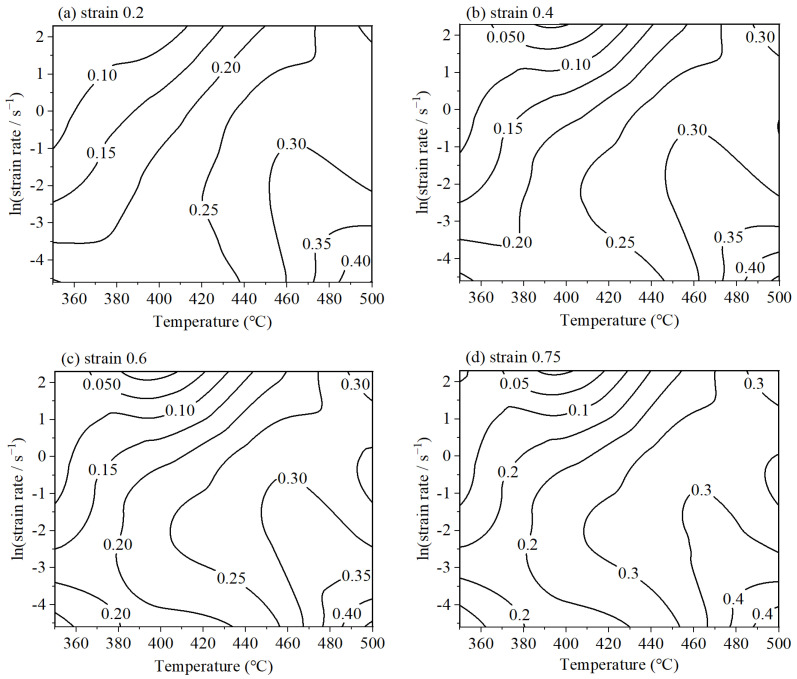
The power dissipation map of 6063: (**a**) strain 0.2; (**b**) strain 0.4; (**c**) strain 0.6; and (**d**) strain 0.75.

A two-dimensional dissipation map can be constructed to represent the power dissipation profile. Figure 7 illustrates the projected power dissipation maps for 6063 alloy under various strain conditions. The map is annotated with contour lines that correspond to different levels of dissipation efficiency, thereby facilitating the visualization of energy distribution during processing. A correlation is established between dissipation efficiency and the mechanisms of microstructural evolution. Notably, the map reveals instances where the dissipation efficiency exceeds 0.4, suggesting highly effective energy utilization. While a higher dissipation efficiency is generally desired for improved material properties, it is worth noting, areas with high dissipation can also be susceptible to the formation of microstructural defects. Consequently, to ensure the desirable mechanical properties, it is imperative to avoid selecting processing parameters that fall within zones of flow instability.

#### 4.3.3. The Instability Parameter ξ

By employing Equations (7) and (9), it is possible to derive a mathematical expression for the instability parameter *ξ*, which is pivotal for assessing the potential for flow instability during material deformation processes [37].

(10) $$\xi (\dot{\varepsilon })=\frac{6d\ln\dot{\varepsilon }+2c}{m(m+1)\ln(10)}+m\leq 0$$

**Figure 8:**
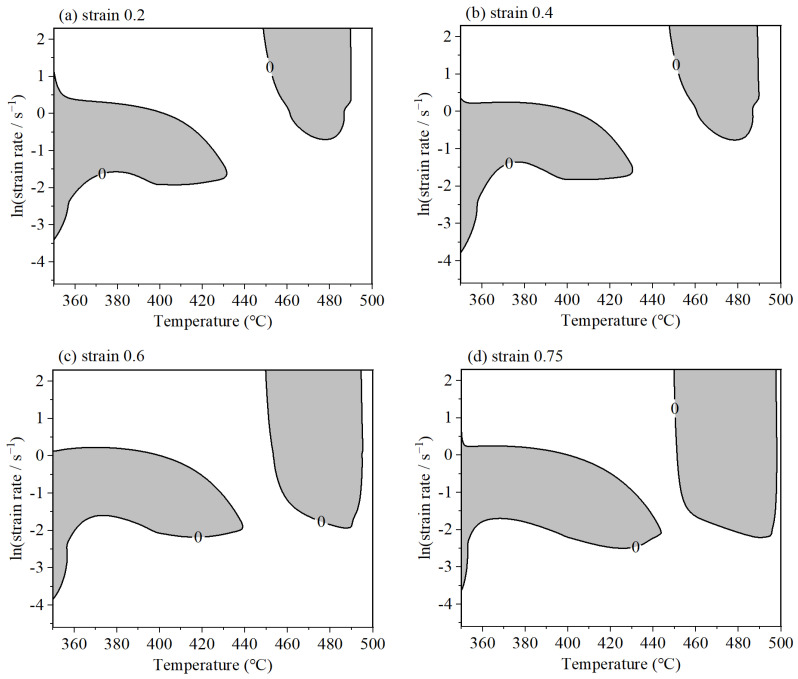
Instability map of 6063 under different deformation conditions. (**a**) Strain 0.2; (**b**) strain 0.4; (**c**) strain 0.6; (**d**) strain 0.75.

With the application of Equation (10), the ξ is deduced, which is essential for mapping the regions of potential flow instability. However, it should be emphasized that during the calculation procedure of ξ, the deformation process is assumed to be steady. In the actual hot working process, complex loading conditions are frequently adopted, which signifies that the calculated ξ in different parameters may exhibit a fluctuating trend, and there is a slight discrepancy with the practical condition. Figure 8 presents the resulting instability maps, which reveal the presence of two distinct instability domains when the strain attains a value of 0.2. These domains are shown within the conditions of (350–430 °C, 0.0334–2.7183 s^−1^) and (450–490 °C, 0.7788–10 s^−1^). While the shape of instability domains remains consistent across various strains, the extent of these regions varies. Upon increasing the strain from 0.2 to 0.75, there is a progressive enlargement of the instability regions, with the corresponding areas expanding to 23.51%, 24.34%, 31.75%, and 36.08%, respectively.

#### 4.3.4. The Processing Map

**Figure 9:**
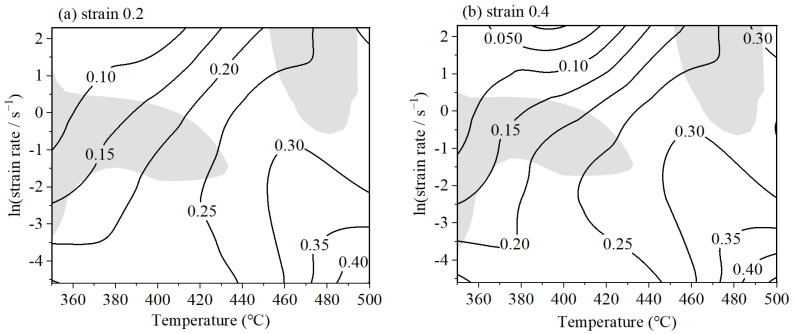

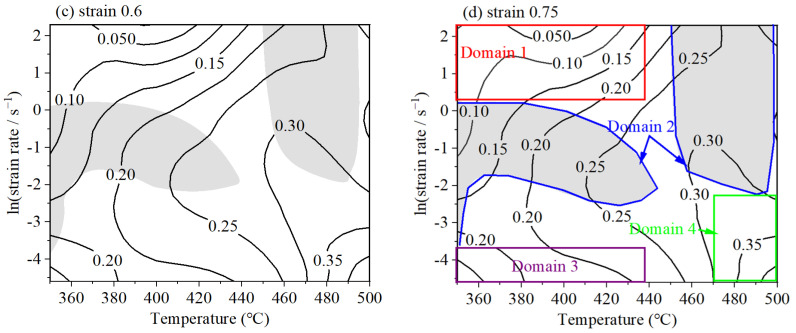
The processing maps of 6063 under different strains: (**a**) 0.2; (**b**) 0.4; (**c**) 0.6; and (**d**) 0.75.

A power dissipation map was constructed to identify the regions with high energy dissipation. Concurrently, an instability map was formulated to describe areas that are susceptible to flow instability. Integrating the information obtained from both these maps, a comprehensive processing map was formulated [38]. Figure 9 presents the processing maps for the 6063 alloy across a range of strains, spanning from 0.2 to 0.75. The contour lines on the map denote the efficiency of power dissipation, while the gray-shaded regions signify domains with negative instability parameters, thereby demarcating the unstable regions.

It is clearly displayed in Figure 9 that the highest power dissipation efficiencies are located within the region characterized by high temperatures and low strain rates, specifically the lower right quadrant of the processing map. These maximum dissipation efficiencies surpass the threshold of 0.4 across the range of strains examined. Upon reaching a true strain of 0.75, as illustrated in Figure 9d, the map delineates four separate domains. Among these, three parts are classified as stable, while one is marked as unstable. Domain 2 is designated as the unstable region, encompassing the parameters (350–443 °C, 0.0302–1.2840 s^−1^) and (450–497 °C, 0.0821–10 s^−1^). Within domain 1, the dissipation efficiency varies from 0.05 to 0.2, signifying a stable zone. Domain 3 is observed to have a high dissipation efficiency, increasing to 0.2; Therefore, it is recognized as a stable zone. Domain 4 is distinguished by its highest power dissipation efficiency values, ranging from 0.3 to 0.4, occurring within the parameters (470–500 °C, 0.01–0.1108 s^−1^), and is identified as the optimal processing zone.

### 4.4. Microstructure Observation

**Figure 10:**
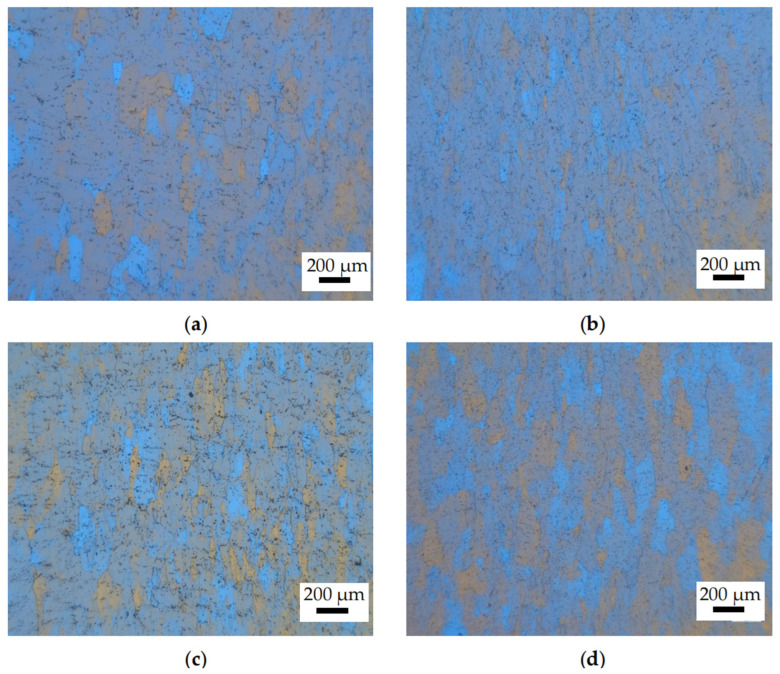
The OM observation of 6063. (**a**) 350 °C 10 s^−1^; (**b**) 350 °C 0.01 s^−1^; (**c**) 500 °C 10 s^−1^; (**d**) 500 °C 0.01 s^−1^.

Figure 10 presents optical microscopy (OM) images of the 6063 aluminum alloy under various conditions. As presented in Figure 10a,b, at 350 °C/10 s^−1^ and 350 °C/0.01 s^−1^ (located in Domain 1 and Domain 3 of Figure 9d), the grains of the 6063 aluminum alloy undergo shape changes under applied force. The equiaxed grains extend in the rolling direction without the observation of voids, cracks, and shear bands. There is no evidence of recrystallized grains, indicating that the DRV softening mechanism has taken place in this domain. The reason is that at lower temperatures, the resistance to dislocation movement becomes higher. Overcoming the potential barrier requires thermal activation, which provides the driving force for dislocation movement to overcome the barrier. However, the thermal activation available at lower temperatures may be insufficient, and the precipitate impedes dislocation movement and increases the resistance, which results in slower movement. This is in agreement with the findings of Xia [37]. As illustrated in Figure 10c, as the temperature increases to 500 °C/10 s^−1^ (located in Domain 2), the density of dislocations increases with the strain rate, and the stored deformation energy becomes more substantial. The serrated grain boundaries begin to multiply, and a trend towards recrystallization is initiated—although it is not clear—and the degree of recrystallization is very low. This is in accordance with the study of Yi [31], where the grains are produced on jagged grain boundaries. In Figure 10d, as the deformation parameter is 500 °C/0.01 s^−1^ (located in Domain 4), grain boundaries begin to partially bend, and fine recrystallized grains were found at the grain boundary junctions. The reason is that the higher deformation temperature provides thermal activation that facilitates dislocation movement. The subgrains become more polygonal and increase in number, leading to a higher nucleation rate and easier recrystallization. This can be explained that the lower deformation rate allows for sufficient deformation time. This provides dislocations enough time to move past equilibrium positions, interact with each other, and continuously undergo slip and climb, leading to a lower dislocation density. However, overall, the degree of recrystallization is very low. The reason for this is that aluminum alloys are high stacking fault energy alloys, and dislocation movement is relatively easier compared to low stacking fault energy metals. The deformation stored energy is consumed excessively, and the remaining part is insufficient, leading to insufficient driving force and fewer recrystallized grains. In summary, the softening mechanism of 6063 is mainly DRV. Both high temperatures and low strain rates are in favor of the diffusion of atoms and the movement of dislocations, which are beneficial for the occurrence of DRV and the appearance of DRX.

**Figure 11:**
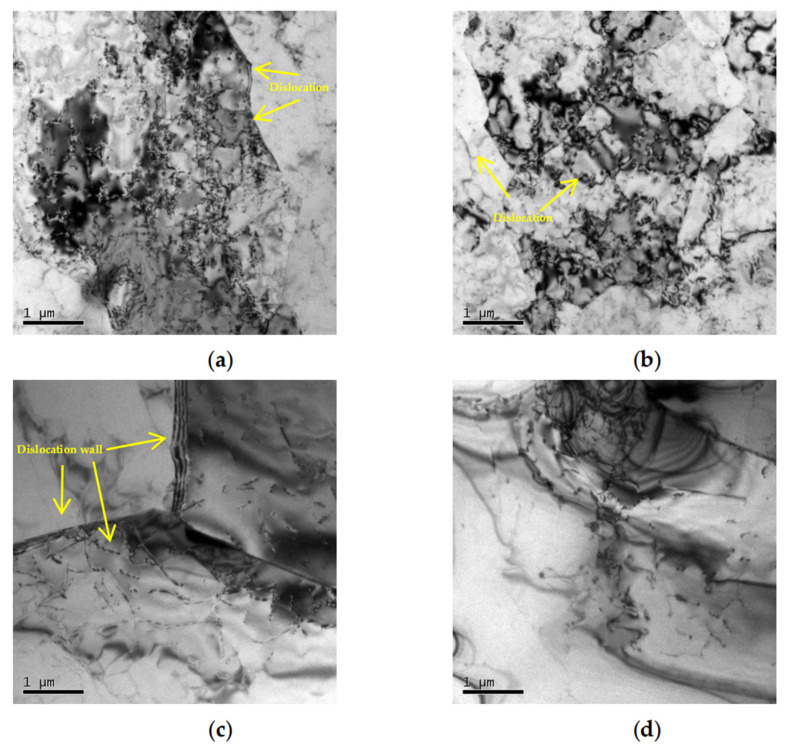
The TEM observation of 6063: (**a**) 0° 350 °C 10 s^−1^; (**b**) 0° 350 °C 0.01 s^−1^; (**c**) 0° 500 °C 10 s^−1^; (**d**) 0° 500 °C 0.01 s^−1^.

The microstructural evolution of the 6063 alloy under different conditions was observed through TEM. Figure 11 shows that at 350 °C/10 s^−1^ (Domain 1), the compressed samples exhibited grains with a high density of dislocation, as shown in Figure 11a. At relatively lower deformation temperatures, the precipitates can pin the dislocations and impede the motion of dislocation; the driving force for dislocation movement induced by deformation is insufficient, leading to the accumulation and entanglement of dislocations, and the corresponding flow stress remains at a relatively high level [39,40,41]. When the deformation rate is decreased to 350 °C/0.01 s^−1^ (Domain 3), as displayed in Figure 11b, the strain rate decreases, allowing enough time for recovery processes within the matrix. Consequently, the dislocation density undergoes a decline; this trend is similar to the findings of Ghosh’s work [16], which is beneficial for the material’s thermal deformation. When fine precipitates are present, the DRX process is hindered by the migration of dislocations and grain boundaries [42]. As the deformation temperature increases, under the condition of 500 °C/10 s^−1^, as observed in Figure 11c, dislocations are significantly reduced. The mobility of dislocations is enhanced due to the temperature rise; the dislocation walls are formed through slip and climb. This phenomenon is in agreement with the observation of Li’s work [43], where the dislocations are in favor of accelerating the DRV rate through climbing and cross-sliding. Meanwhile, as the temperature increases, many precipitates dissolve into the matrix, the dislocations and vacancies around the precipitates play a role in diffusion [44], and the density of precipitates decreases. Additionally, there are still some areas with high dislocation density. At high strain rates, the time for the dislocations to migrate and annihilate is limited, leading to the accumulation of dislocations and stress concentration. During the hot processing, defects are prone to forming in that region. Therefore, under high strain rates, dislocations tend to accumulate, which may be stress concentration points and initiate cracks in Domain 2. The material may undergo failure with a short serving life. It is evident that Domain 2 is an unstable region. As illustrated in Figure 11d, as the strain rate declines to 0.01 s^−1^ (Domain 4), there is a downward trend in dislocation density. At lower strain rates, dislocation movement occurs over a sufficient time, allowing for the rearrangement and annihilation of dislocations. This is beneficial for reducing stress concentration and forming a stable microstructure. Consequently, within this domain, a finer-grain structure can be achieved; this observation has also been reported by Xu [42]. At the same time, precipitates are seldom observed, and the density of dislocation remains at a low level. Although only one set of thermomechanical parameters was selected for each domain, the microstructural analysis correlates well with the processing map, thereby proving the availability of the processing map. It is clear that the appropriate thermomechanical processing domain at a strain of 0.75 is (470–500 °C, 0.01–0.1108 s^−1^), with a corresponding peak efficiency of 0.4.

## 5. Conclusions

Hot compression was tested on 6063 aluminum alloy across a range of temperatures (350, 400, 450, and 500 °C) and medium strain rates (0.01, 0.01, 1, and 10 s^−1^) to evaluate its hot workability. This study employed a processing map and microstructural examination to analyze the alloy’s behavior under various thermal and mechanical conditions. Key findings are concluded as follows:

1. The flow stress of the 6063 aluminum alloy increases significantly with rising strain rate and declines with increasing deformation temperatures during hot compression. A set of hot processing maps was established, and the optimal hot working conditions for the 6063 aluminum alloy at an applied strain of 0.75 were identified within the temperature and strain rate range (470–500 °C, 0.01–0.1108 s^−1^), achieving a peak efficiency of 0.4.
2. During hot compression, the equiaxed grains elongate in the vertical direction of compression, and no voids or cracks are present. The density of dislocation increases with increasing strain rate and declining temperature. The main softening mechanism of the alloy is dynamic recovery. The microstructural observations align closely with the processing map outcomes.

## Figures and Tables

**Table 1:** The chemical composition of 6063 alloy.

Composition	Mg	Cu	Si	Cr	Fe	Zn	Mn	Ti	Al
Content (wt.%)	0.65	0.1	0.46	0.1	0.18	0.1	0.05	0.08	Bal.

## Data Availability

The original contributions presented in this study are included in the article. Further inquiries can be directed to the corresponding author.

## References

[1] Li S.S., Yue X., Li Q.Y., Peng H., Dong B., Liu T., Yang H., Fan J., Shu S., Qiu F. (2023). Development and applications of aluminum alloys for aerospace industry. J. Mater. Res. Technol..

[2] Lv Q., Zhang F., Wei H., Li Z., Zhang J. (2023). Effect of rare earth (La, Ce, Nd, Sc) on strength and toughness of 6082 aluminum alloy. Vacuum.

[3] Tenali N., Ganesan G., Babu P.R. (2024). An investigation on the mechanical and tribological properties of an ultrasonic-assisted stir casting Al-Cu-Mg matrix-based composite reinforced with agro waste ash particles. Appl. Eng. Lett..

[4] Sharma S.K., Gajević S., Sharma L.K., Pradhan R., Sharma Y., Miletić I., Stojanović B. (2024). Progress in Aluminum-Based Composites Prepared by Stir Casting: Mechanical and Tribological Properties for Automotive, Aerospace, and Military Applications. Lubricants.

[5] Chen G., Liu J., Dong Z., Shu X., Zhang B. (2020). Underlying reasons of poor mechanical performance of thick plate aluminum-copper alloy vacuum electron beam welded joints. Vacuum.

[6] Kumar N. (2022). Severe Plastic Deformation of Al–Mg–Si Alloys Processed Through Rolling Techniques: A Review. Metallogr. Microstruct. Anal..

[7] Han Y., Shao D., Chen B.A., Peng Z., Zhu Z.X., Zhang Q., Chen X., Liu G., Li X.M. (2017). Effect of Mg/Si ratio on the microstructure and hardness–conductivity relationship of ultrafine-grained Al-Mg-Si alloys. J. Mater. Sci..

[8] Omotoyinbo J.A., Oladele I.O. (2010). The effect of plastic deformation and magnesium content on the mechanical properties of 6063 aluminium alloys. J. Miner. Mater. Charact. Eng..

[9] Siddiqui R.A., Abdullah H.A., Al-Belushi K.R. (2000). Influence of aging parameters on the mechanical properties of 6063 aluminium alloy. J. Mater. Process. Technol..

[10] Lu R., Zheng S., Teng J., Hu J., Fu D., Chen J., Zhao G., Jiang F., Zhang H. (2021). Microstructure, mechanical properties and deformation characteristics of Al-Mg-Si alloys processed by a continuous expansion extrusion approach. J. Mater. Sci. Technol..

[11] Zhao J., Shi X., Liu M., Liu Z., Luo B., Yu K., Li Q., Ran J., Zhao P. (2024). Effect of hot rolling finishing temperature and intermediate annealing on the microstructure, texture evolution, and comprehensive properties of Al-Mg-Si alloy. J. Alloys Compd..

[12] Zhao N., Ma H., Hu Z., Yan Y., Chen T. (2022). Microstructure and mechanical properties of Al-Mg-Si alloy during solution heat treatment and forging integrated forming process. Mater. Charact..

[13] Prasad Y., Seshacharyulu T. (1998). Modelling of hot deformation for microstructural control. Int. Mater. Rev..

[14] Prasad Y., Rao K.P. (2011). Materials modeling and finite element simulation of isothermal forging of electrolytic copper. Mater. Des..

[15] Yu Y., Pan Q., Wang W., Huang Z., Xiang S., Liu B. (2021). Dynamic softening mechanisms and Zener-Hollomon parameter of Al–Mg–Si–Ce–B alloy during hot deformation. J. Mater. Res. Technol..

[16] Ghosh A., Elasheri A., Parson N., Chen X.-G. (2024). Hot deformation behavior and processing maps for an Al-Mg-Si-Zr-Mn Alloy. J. Alloys Metall. Syst..

[17] Feng X., Wang Y., Huang Q., Liu H., Zhang Z. (2024). The dynamic recrystallization microstructure characteristics and the effects on static recrystallization and mechanical properties of Al–Mg–Si alloy. Mater. Sci. Eng. A.

[18] Ke B., Ye L., Tang J., Zhang Y., Liu S., Lin H., Dong Y., Liu X. (2020). Hot deformation behavior and 3D processing maps of AA7020 aluminum alloy. J. Alloys Compd..

[19] Miao J., Sutton S., Luo A.A. (2022). Deformation microstructure and thermomechanical processing maps of homogenized AA2070 aluminum alloy. Mater. Sci. Eng. A.

[20] Su Y., Kong F., You F., Wang X., Chen Y. (2020). The high-temperature deformation behavior of a novel near-α titanium alloy and hot-forging based on the processing map. Vacuum.

[21] Li J., Wu X., Cao L., Liao B., Wang Y., Liu Q. (2021). Hot deformation and dynamic recrystallization in Al-Mg-Si alloy. Mater. Charact..

[22] Qu S., Li Y. (2024). Characterization of Hot Deformation Behavior and Processing Maps of Homogenized Al-Mg-Si-Sc-Zr Alloys. J. Mater. Eng. Perform..

[23] Muhammad M., Nezhadfar P., Thompson S., Saharan A., Phan N., Shamsaei N. (2021). A comparative investigation on the microstructure and mechanical properties of additively manufactured aluminum alloys. Int. J. Fatigue.

[24] Cao H., Sun Q., Pu Q., Wang L., Huang M., Luo Z., Che J. (2020). Effect of vacuum degree and T6 treatment on the microstructure and mechanical properties of Al–Si–Cu alloy die castings. Vacuum.

[25] Snopiński P., Król M., Tański T., Pakuła D., Kříž A. (2021). Effect of initial microstructure on hot deformation behavior of AlMg5Si2Mn alloy. Mater. Charact..

[26] Jin D., Li H., Zhu Z., Yang C., Chen B., Miao Y. (2023). Effects of cooling rate in homogenization on microstructure, hot deformation resistance and subsequent age-hardening behavior of an Al–Mg–Si alloy. J. Mater. Res. Technol..

[27] Zhao Q., Li F., Zhu E., Gopi K.R., Farah S., An X., Yao K., Li J., Hashmi A.F., Liu L. (2024). Investigation on the grain structure evolution and abnormal stress increase of Al–Mg–Si alloy during hot deformation. Met. Mater. Int..

[28] Hu M., Sun Y., He J., Li C., Li H., Yu L., Liu Y. (2022). Hot deformation behaviour and microstructure evolution of Al-3% Mg2Si alloy. Mater. Charact..

[29] Mirahmadi D., Dehghani K., Shamsipur A., Kalaki A. (2023). Hot deformation behavior, microstructure evolution and processing map of Cu–2Be alloy. J. Mater. Res. Technol..

[30] Volodko S., Yudin S., Korotitskiy A., Markova G., Cheverikin V., Permyakova D., Poliakov M., Titov D., Moskovkikh D., Kasimtsev A. (2024). Hot deformation behavior of NiTiHf alloy under compression: Effect of deformation heating on flow softening. Mater. Charact..

[31] Yi H., Ding J., Ni C., Dai J., Tang Y., Chen X., Song K., Xia X. (2022). Hot compression deformation behavior and processing maps of Al-0.5 Mg-0.4 Si-0.1 Cu alloy. J. Mater. Res. Technol..

[32] Zhao N., Sun Q., Pang Q., Hu Z. (2023). Comprehensive study of hot compression behaviors and microstructure evolution of solutionized 6082 aluminum alloy extruded bar. J. Alloys Compd..

[33] Prasad Y., Seshacharyulu T. (1998). Processing maps for hot working of titanium alloys. Mater. Sci. Eng. A.

[34] Wang H., Wang C., Mo Y., Wang H., Xu J. (2019). Hot deformation and processing maps of Al–Zn–Mg–Cu alloy under coupling-stirring casting. J. Mater. Res. Technol..

[35] Chamanfar A., Alamoudi M.T., Nanninga N.E., Misiolek W.Z. (2019). Analysis of flow stress and microstructure during hot compression of 6099 aluminum alloy (AA6099). Mater. Sci. Eng. A.

[36] Tang J., Wang J., Teng J., Wang G., Fu D., Zhang H., Jiang F. (2021). Effect of Zn content on the dynamic softening of Al–Zn–Mg–Cu alloys during hot compression deformation. Vacuum.

[37] Xia E., Ye T., Qiu S., Liu L., Luo F., Yue H., Wu Y. (2023). Deformation Behavior and Processing Maps of 7075 Aluminum Alloy under Large-Strain Thermal Compression. Materials.

[38] Wang J., Xiao G., Zhang J. (2023). A new constitutive model and hot processing map of 5A06 aluminum alloy based on high-temperature rheological behavior and higher-order gradients. Mater. Today Commun..

[39] Deng L., Zhang H.-D., Li G.-A., Tang X.-F., Yi P.-S., Liu Z., Wang X.-Y., Jin J.-S. (2022). Processing map and hot deformation behavior of squeeze cast 6082 aluminum alloy. Trans. Nonferrous Met. Soc. China.

[40] Zhao H., Ye L., Cheng Q., Kang Y., Zhang W. (2022). Constitutive model and processing maps of 7055 aluminum alloy used for fasteners. Mater. Today Commun..

[41] Liu W., Man Q., Li J., Liu L., Zhang W., Wang Z., Pan H. (2022). Microstructural evolution and vibration fatigue properties of 7075-T651 aluminum alloy treated by nitrogen ion implantation. Vacuum.

[42] Xu C., Huang J., Jiang F., Jiang Y. (2022). Dynamic recrystallization and precipitation behavior of a novel Sc, Zr alloyed Al-Zn-Mg-Cu alloy during hot deformation. Mater. Charact..

[43] Li H., Huang Y., Liu Y. (2023). Dynamic recrystallization mechanisms of as-forged Al–Zn–Mg-(Cu) aluminum alloy during hot compression deformation. Mater. Sci. Eng. A.

[44] Sun D., Zhang X., Ye L., Gu G., Jiang H., Gui X. (2015). Evolution of θ′ precipitate in aluminum alloy 2519A impacted by split Hopkinson bar. Mater. Sci. Eng. A.

